# RF Sensing Based Breathing Patterns Detection Leveraging USRP Devices

**DOI:** 10.3390/s21113855

**Published:** 2021-06-02

**Authors:** Mubashir Rehman, Raza Ali Shah, Muhammad Bilal Khan, Najah Abed AbuAli, Syed Aziz Shah, Xiaodong Yang, Akram Alomainy, Muhmmad Ali Imran, Qammer H. Abbasi

**Affiliations:** 1 Department of Electrical Engineering, HITEC University, Taxila 47080, Pakistan; 18-phd-ee-002@student.hitecuni.edu.pk (M.R.); raza.ali.shah@hitecuni.edu.pk (R.A.S.); 2 Department of Electrical and Computer Engineering, Attock Campus, COMSATS University Islamabad, Attock 43600, Pakistan; bilal@stu.xidian.edu.cn; 3 School of Electronic Engineering, Xidian University, Xi’an 710071, China; xdyang@xidian.edu.cn; 4 College of Information Technology, United Arab Emirates University (UAEU), Al-Ain, United Arab Emirates; 5 Research Centre for Intelligent Healthcare, Coventry University, Coventry CV1 5FB, UK; syed.shah@coventry.ac.uk; 6 School of Electronic Engineering and Computer Science, Queen Mary University of London, London WC1E 7HU, UK; a.alomainy@qmul.ac.uk; 7 School of Engineering, University of Glasgow, Glasgow G12 8QQ, UK; Muhammad.Imran@glasgow.ac.uk (M.A.I.); Qammer.Abbasi@glasgow.ac.uk (Q.H.A.); 8 Artificial Intelligence Research Centre (AIRC), Ajman University, Ajman, United Arab Emirates

**Keywords:** CSI, OFDM, SDR, USRP, breathing pattern, COVID-19

## Abstract

Non-contact detection of the breathing patterns in a remote and unobtrusive manner has significant value to healthcare applications and disease diagnosis, such as in COVID-19 infection prediction. During the epidemic prevention and control period of COVID-19, non-contact approaches have great significance because they minimize the physical burden on the patient and have the least requirement of active cooperation of the infected individual. During the pandemic, these non-contact approaches also reduce environmental constraints and remove the need for extra preparations. According to the latest medical research, the breathing pattern of a person infected with COVID-19 is unlike the breathing associated with flu and the common cold. One noteworthy symptom that occurs in COVID-19 is an abnormal breathing rate; individuals infected with COVID-19 have more rapid breathing. This requires continuous real-time detection of breathing patterns, which can be helpful in the prediction, diagnosis, and screening for people infected with COVID-19. In this research work, software-defined radio (SDR)-based radio frequency (RF) sensing techniques and machine learning (ML) algorithms are exploited to develop a platform for the detection and classification of different abnormal breathing patterns. ML algorithms are used for classification purposes, and their performance is evaluated on the basis of accuracy, prediction speed, and training time. The results show that this platform can detect and classify breathing patterns with a maximum accuracy of 99.4% through a complex tree algorithm. This research has a significant clinical impact because this platform can also be deployed for practical use in pandemic and non-pandemic situations.

## 1. Introduction

During the breathing mechanism, oxygen is inhaled, and carbon dioxide is exhaled to tightly regulate the partial pressures of oxygen and carbon dioxide in arterial blood. This mechanism is accomplished by setting the respiratory rate and tidal volume of the human body [1]. The number of respiratory cycles per minute represents the respiratory or breathing rate and is considered one of the four primary vital signs of human life. The vital signs are measurements of basic functions of the human body [2]. The rate and depth of breathing are automatically controlled by numerous body mechanisms, which maintain the consistency of the partial pressures of oxygen and carbon dioxide in the arterial blood. The normal breathing rate for an adult at rest varies from 12–20 breaths/min, and it is considered abnormal if this rate is under 12 breaths/min or over 20 breaths/min [3]. In the human body, breathing has many other significant functions. It provides a mechanism for talking, laughter, and similar expressions of emotions. Breathing is also used for human reflexes, for example, coughing, sneezing, and yawning. Breathing is accomplished mainly through the contraction of the diaphragm and the intercostal muscles, which pull the rib cage outwards and upwards [4]. Breathing through the diaphragm is called diaphragmatic breathing, which causes the abdomen to regularly expand and contract. It is therefore also named abdominal breathing or deep breathing. When breathing is achieved through intercostal muscles, then it is called costal breathing or shallow or chest breathing [5].

**Figure 1:**
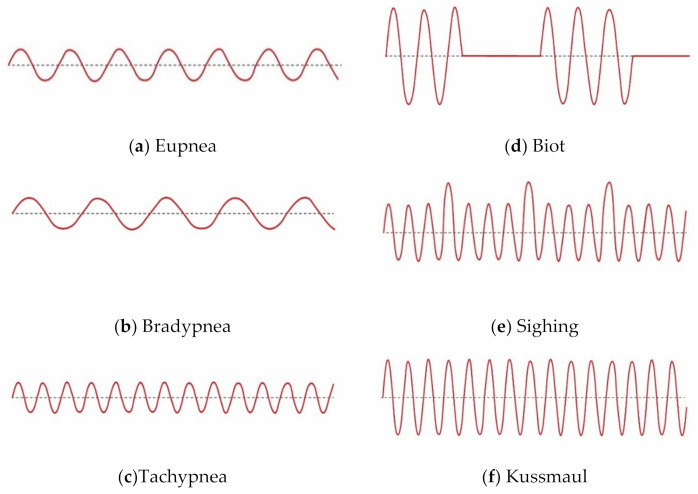
Different breathing patterns.

Breathing is not only a process of inhaling and exhaling air. The entire respiratory pattern is vital to human health. Depth, rate, timing, and consistency of breaths are all significant for the balance of respiration and metabolism. This is the reason breathing is considered important for human life, and useful in diagnosis and monitoring health issues. Abnormalities in breathing are frequently caused by injury to respiratory centres in pons and medulla, use of narcotic medications, metabolic derangements, and respiratory muscle weakness [1]. Abnormal breathing patterns may also indicate the potential for injury or metabolic illnesses, and these patterns may also reflect emotional imbalance [6] and stress [7]. Several clinical studies propose that abnormal breathing patterns can forecast specific diseases [8], thus providing comprehensive evidence for medical treatment [9]. Therefore, vigilant observation of the breathing rate and pattern is critical in the analysis and treatment of numerous diseases [10]. Due to different medical conditions, such as metabolic illness or potential injury, breathing can lose its normal rhythm. Abnormalities in breathing patterns may be due to incorrect use of muscles to breathe, use of the upper chest instead of the diaphragm, and mouth breathing instead of nose breathing. Abnormal breathing patterns can be slow, fast, shallow, or deep, or a combination of these breaths. The different breathing patterns include eupnea, bradypnea, tachypnea, biot, sighing, and kussmaul [11], as shown in Figure 1. Eupnea is breathing with a normal pattern and rate, bradypnea is slow and shallow breathing; whereas, tachypnea is the opposite of bradypnea, i.e., it is fast and shallow breathing. Biot is a deep breath with gradual periods of no breaths, sighing is breathing punctuated by frequent deep breathes, whereas kussmaul is a fast and deep breath. The descriptions and causes of these different abnormal breathing patterns are given in Table 1.

The novel coronavirus disease (COVID-19) is occurred by the Severe Acute Respiratory Syndrome Coronavirus 2 (SARS-CoV-2) virus [12] and mainly causes a lower respiratory tract infection. A severe attack of this disease can cause intensive respiratory failure [13]. The containment of COVID-19 is challenging and dangerous due to its high transmission during the pre-symptomatic incubation phase and widespread shortage of testing. In addition to conventional laboratory testing, other schemes have been proposed for COVID-19 monitoring. These schemes include social distancing and day-to-day monitoring of temperature to detect and isolate possibly infected people. These methods can be efficient in detecting infected people who are showing symptoms such as fever; however, fever-based screening fails for cases of infected people without symptoms. This limitation of fever-based screening is noteworthy and suggests there another screening tool is necessary. Breathing rate monitoring may become a common COVID-19 screening tool to detect lower respiratory tract infections in medical research. Because COVID-19 damages the respirational system, it is sensible to propose that the variations in breathing might occur during the early stages of COVID-19 infection. In this context, non-contact regular monitoring of breathing can be used to identify intraindividual breathing variations and detect potential infections that could be overlooked by clinical thresholds [14]. If variations in breathing are found to be a precise indicator of COVID-19 infection, then breathing monitoring could be a protocol used by medical specialists and administrations to impose self-isolation and target testing. Unfortunately, these abnormal breathing patterns occur in such a manner that they are difficult to detect by the patients themselves. The development of a system to detect these abnormal breathing patterns remotely and efficiently under several scenarios could help in the prediction of COVID-19 infection, in addition to assisting individuals to diagnose various breathing diseases at the earliest stage. Thus, long-term and real-time monitoring of breathing could be used in the diagnosis of COVID-19 infection and various other breathing disorders. Hence, there is a need for a non-contact method that can precisely detect different breathing patterns due to various medical conditions.

Diverse technologies and techniques are reported in the literature for the examination and classification of different breathing patterns. The finest technique in hospitals is spirometry, which directly determines the air volume and flows during inhalation and exhalation [15]. Other respiration techniques used in hospitals consist of inductance pneumography [16], electrical impedance pneumography (EIP) [17], and capnography [18]. However, these techniques require patients to visit the hospital. The other technologies for breathing pattern detection are mainly divided into two categories: contact-based or non-contact. Contact-based breathing measurement devices are heavy, expensive, and often cause inconvenience for patients to use [19]. Therefore, non-contact measurement technologies are more appropriate for detecting breathing patterns. These non-contact measurements may include camera-based sensing and RF sensing. Camera-based sensing may use a thermal imaging camera or depth camera [20]. Both of these camera-based technologies have limitations; for example, thermal imaging is susceptible to ambient heat [21], whereas depth cameras are expensive and have a high computational cost. RF sensing for breathing is further divided into different technologies, such as radar, Wi-Fi, or SDR. These technologies may use received signal strength (RSS) or channel state information (CSI) for RF signal sensing. CSI is considered more stable compared to RSS because it provides fine-grained information. Radar-based RF sensing is a non-contact solution; however, this requires specialized devices with high complexity, and frequent use of radar has the potential hazard associated with the released radiation [22,23]. Wi-Fi-based RF sensing can also be based on RSS, CSI, and frequency modulated continuous waves (FMCWs) [24,25,26]. Wi-Fi-based RF sensing has various advantages because it is a cost-effective solution and hardware is easily available; however, it also has limitations, such as lack of flexibility and scalability, and under-reporting of subcarriers [27]. Similarly, the S-band sensing technique is used to detect human breathing and other abnormalities in [28,29,30,31,32]. Among these, SDR-based RF sensing for breathing detection is most effective and efficient because it provides a flexible, scalable, and portable solution. This method also allows custom configuration of transmitted and received power and the selection of the operating frequency. In addition, it offers easy implementation of signal processing algorithms. Machine learning (ML) is also used in the area of breathing detection because it can help in the accurate classification of different breathing patterns. Many authors have used ML for breathing pattern classification [33]. In these previous studies, some authors failed to obtain suitable accuracy, and some classified only basic breathing patterns, such as fast and normal breathing [34]. Therefore, there is a need for a platform that can not only detect but also accurately classify different breathing patterns.

This research work contributes to the development of a non-contact SDR-based RF sensing platform. This study aimed to detect and classify different breathing patterns for COVID-19 and non-COVID-19 scenarios. The developed platform has the ability to detect and classify different breathing patterns relevant to certain diseases in an indoor environment. This platform leverages the readily available CSI to detect slight changes in the environment caused by different types of breathing, and provides a flexible, portable solution. This SDR-based platform also permits modification of numerous parameters, such as the number of frequency carriers and the power level. Fine-grained CSI containing amplitude information of multiple orthogonal frequency division multiplexing (OFDM) subcarriers is utilized for real-time and long-term monitoring of different breathing patterns.

The rest of the paper is organized as follows. Section 2 describes the system design of the non-contact platform to detect breathing patterns. In Section 3, a detailed methodology of the experiments conducted for the development of the machine learning model is explained. Section 4 shows the results and discusses the various breathing patterns detected by the non-contact SDR-based platform. Finally, conclusions drawn from this research work and future recommendations are presented in Section 5.

## 2. System Design

**Figure 2:**
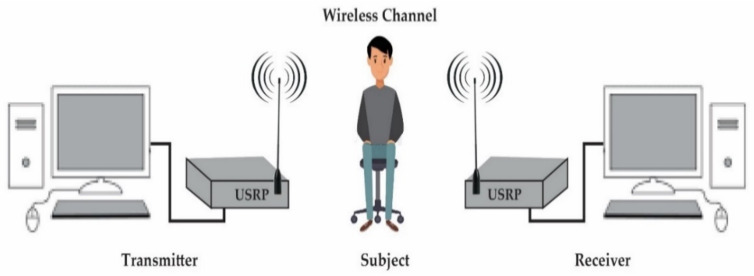
System design.

The system design consists of PCs to enable the software functionality of SDR in LabVIEW. A universal software radio peripheral (USRP) model 2922 was used for implementing the generic RF functionality of SDR technology, and omnidirectional antennas were used to capture the CSI. This system is used to detect and classify different breathing patterns by observing small-scale movements in the wireless channel via gathering fine-grained CSI. The RF signal generated by the transmitter in the indoor environment reaches the receiver via multipath. This received signal contains information about environmental characteristics. In this context, the environment is considered to be the physical space containing human factors, such as human position and breathing style, and environmental features [35]. When a person is present in the physical space, an additional path exists due to the reflection or diffraction of signals from the person’s body. Therefore, the effect of human movement on the propagation of signals is recorded by the received signals and described in the form of CSI. Later, information retrieved from CSI can be used to detect different breathing patterns. In this platform, the transmitter USRP continuously transmits wireless signals with a specific frequency, and the receiver USRP receives these signals. Minute variations in the chest and abdomen due to breathing activity result in a change in the signal propagation path recorded by the received signals in the form of CSI. This non-contact SDR-based platform consists of three major functional blocks including transmitter, wireless channel, and receiver, as shown in Figure 2.

### 2.1. Transmitter

The transmitter consists of a transmitter PC and transmitter USRP. In the transmitter PC, pseudo-random (PN) data bits are produced and mapped to quadrature amplitude modulation (QAM) symbols. These symbols are divided into parallel streams. Then, reference data symbols are concatenated in each parallel frame. These reference symbols are beneficial on the receiver side for channel estimation. Zeros are placed at edges, and one zero at DC in every frame. After zero padding, an inverse fast Fourier transform (IFFT) operation is applied to convert frequency-domain signals to time-domain signals. A cyclic prefix (CP) is inserted by duplicating the last one-quarter of points at the beginning of every frame. This insertion of a CP helps at the receiver side in the removal of time and frequency offset. This synthesized data from the host PC is sent to the USRP kit through gigabit ethernet at a rate of 20 MS/s. The USRP hardware interpolates the incoming signal to 400 MS/s using a digital up-conversion (DUC) and then translates the signal to analog using a digital-to-analog converter (DAC). The resultant analog signal is passed through a low-pass filter with a bandwidth of 20 MHz and then mixed to the specified carrier frequency. This signal is then passed through a transmit amplifier, where its gain can be varied between 0 and 30 dB. Then, this signal is transmitted through an omnidirectional antenna.

### 2.2. Wireless Channel

In this platform, an indoor wireless channel is used to collect information about different breathing patterns due to minute human movements during breathing. The CSI signal is composed of multipath signals, which are generated due to human body movements between the two omnidirectional antennas.

### 2.3. Receiver

The signal at the receiver side is first received by the USRP kit through the omnidirectional antenna. After being passed through a low-noise amplifier (LNA), which reduces the noise component, this signal is passed through a drive amplifier (DA) to adjust its gain. The resultant signal is mixed using a direct conversion receiver (DCR) into a baseband complex signal. This signal is passed through a low-pass filter (LPF) with a bandwidth of 20 MHz, which is then sampled at 100 MS/s by a 2-channel analog-to-digital converter (ADC). This digitized complex signal moves to a digital down converter (DDC) that mixes, filters, and decimates this signal to a user-specified rate. Finally, this down-converted signal is passed to the host PC through a gigabit ethernet cable at up to 20 MS/s. The receiver host PC not only removes the CP from each frame but also uses it to remove time and frequency offset using the Van de Beek algorithm [36,37]. After CP removal from each frame, FFT is then applied to convert the time domain OFDM samples to the frequency domain OFDM symbol. Then, the amplitude response of the frequency domain signal is extracted to detect different breathing patterns.

## 3. Methodology

**Figure 3:**
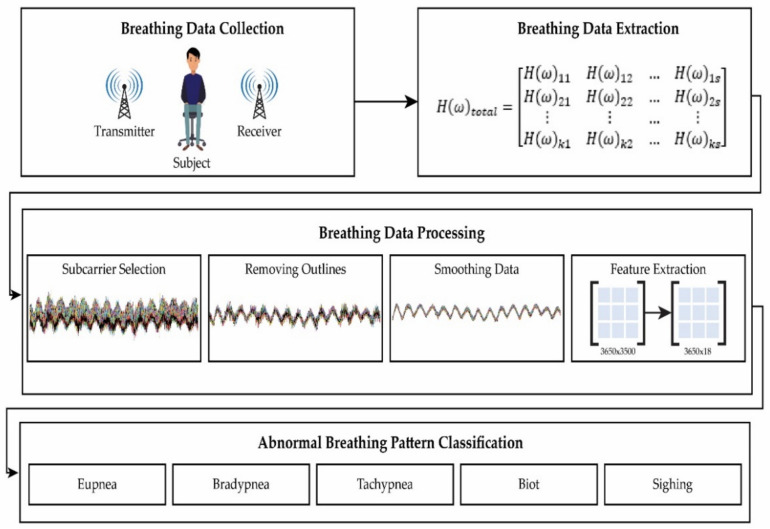
Methodology block diagram.

The various steps involved in the methodology are shown in Figure 3. These steps are given below:

- Breathing data collection;
- Breathing data extraction;
- Breathing data processing;
- Breathing pattern classification.

Each step is explained below in detail.

### 3.1. Breathing Data Collection

Breathing data collection is carried out in a lab environment. The experimental setup consists of two USRPs (NI-2922); the distance between the USRPs and the subject is maintained at 1 m. Each subject was asked to sit on a stool in a relaxed posture with minimal body movements. Both USRPs are placed at the same height, parallel to the abdomen of the subject. A total of five participants were asked to perform different breathing patterns; details of each participant are given in Table 2. Before breathing data collection, subjects were professionally trained to perform each breathing pattern according to medical data. In this research work, each subject was asked to perform six different breathing patterns. For six breathing patterns, five data sets were collected from five subjects and a total of 150 experiments were performed. Each breathing pattern activity was performed for 30 s by each subject. Extensive experimentation was performed to ensure a high level of accuracy.

### 3.2. Breathing Data Extraction

On the receiver side, the received OFDM signal is used for fine-grained CSI extraction. From this received OFDM signal, both amplitude and phase frequency response can be acquired. In this research, however, only amplitude frequency response was used for further processing. The breathing activity is detected following the determination of the amplitude frequency response for each activity. If the amplitude response is close to the actual breathing pattern, as shown in Figure 1, then this amplitude response is accepted; otherwise, it is rejected, and the subject is asked to perform this breathing pattern again more professionally. This amplitude response depicts various information, such as the number of subcarriers and the number of OFDM samples. The number of OFDM samples received depends upon various factors, such as the time taken to perform each breathing activity.

The total frequency response $H\left(j\omega \right)\;$ of each activity is given by Equation (1):

(1) $$H{\left(\omega \right)}_{total}=\left[\begin{array}{cccc} H{\left(\omega \right)}_{11} & H{\left(\omega \right)}_{12} & \ldots & H{\left(\omega \right)}_{1s}\\ H{\left(\omega \right)}_{21} & H{\left(\omega \right)}_{22} & \ldots & H{\left(\omega \right)}_{2s}\\ \vdots & \vdots & \ldots & \vdots \\ H{\left(\omega \right)}_{k1} & H{\left(\omega \right)}_{k2} & \ldots & H{\left(\omega \right)}_{ks} \end{array}\right]$$

where k shows the total number of subcarriers and s shows the total number of samples received when each activity is performed for 30 s. The frequency response contains both amplitude and phase information, which can be expressed in Equations (2) and (3) as:

(2) $$\left|H\left(\omega_{k}\right)\right|=\sqrt{H{\left(\omega_{k}\right)}_{real}{}^{2}+H{\left(\omega_{k}\right)}_{img}{}^{2}}$$

(3) $$∠H\left(\omega_{k}\right)=-tan^{-1}\left(\frac{H{\left(\omega_{k}\right)}_{img}}{H{\left(\omega_{k}\right)}_{real}}\right)$$

In this research, only amplitude response is used for detecting and classifying different breathing patterns.

### 3.3. Breathing Data Processing

Breathing data processing was performed on the data acquired by subject 2. This data processing was divided into four steps:

#### 3.3.1. Subcarrier Selection

**Figure 4:**
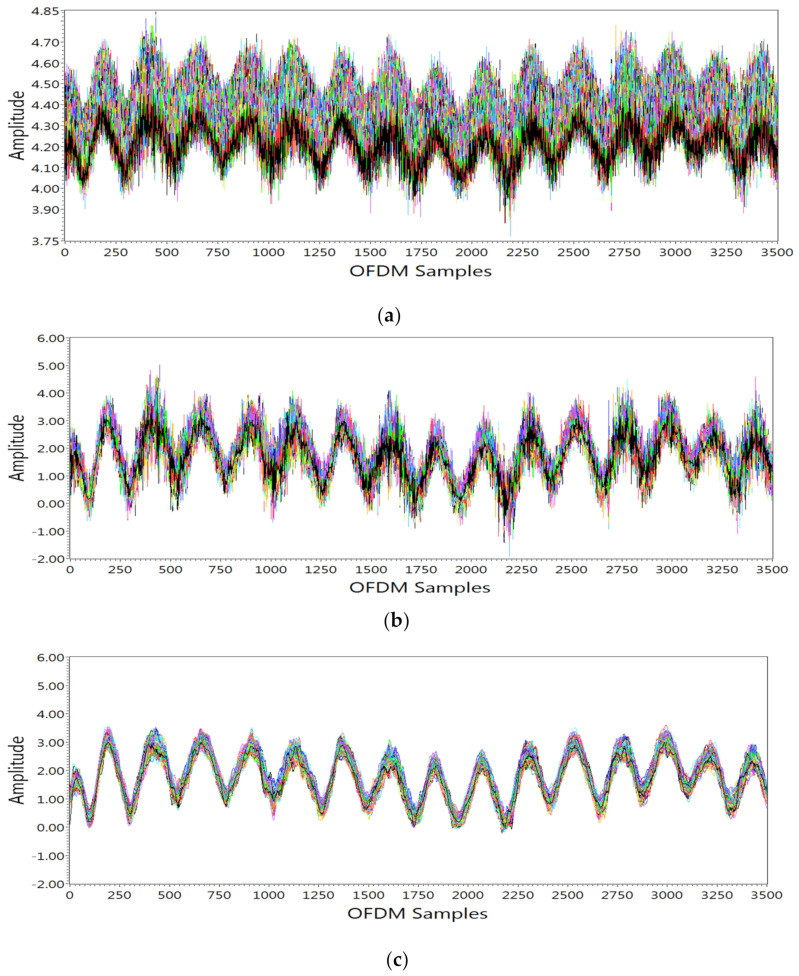
Breathing data processing: (**a**) data after subcarrier selection; (**b**) data after outlier removal; (**c**) data after smoothing.

A group of 256 subcarriers was acquired at the receiver side for each activity. It was observed that the amplitude of each subcarrier shows different sensitivity to breathing activity. For better detection of the breathing pattern, it was necessary to remove all those subcarriers which were less sensitive to breathing activity. The variance of subcarriers was calculated and, on the basis of this, those subcarriers with less sensitivity to breathing activity were rejected, as seen in Figure 4a.

#### 3.3.2. Removing Outliers

After subcarrier selection, wavelet filtering was performed. The wavelet filter not only removes outliers from raw data but also retains sharp transition, as shown in Figure 4b. For wavelet filtering, soft heuristic SURE thresholding was applied with the scaled noise option on detail coefficients by selecting level 4 and syms5 wavelet.

#### 3.3.3. Smoothening Data

To smooth the data and remove high-frequency noise not produced by breathing activity, a moving average filter with window size 8 was used, as shown in Figure 4c. After performing the above operations, different breathing patterns could easily be detected.

#### 3.3.4. Feature Extraction

After processing, breathing data represent CSI regarding different breathing patterns. To obtain meaningful information from data, feature extraction was performed. Feature extraction plays a highly significant role in classification approaches because it reduces computation complexity by decreasing dimension size [38,39]. In this research, 18 statistical features were extracted. After feature extraction, the dimension of data size was reduced to 3650 × 18 from 3650 × 3500. The detail of these features is shown in Table 3.

### 3.4. Classification

The breathing data after feature extraction was used for the classification of different breathing patterns using ML algorithms. Various algorithms were exploited for this purpose. The accuracy and efficiency of ML algorithms are dependent on the type and size of the data set. Different algorithms result in different performance levels for the different data sets, and the efficiency of algorithms increases by increasing the size of the dataset. Random 5-fold cross-validation was used for breathing pattern classification.

## 4. Results and Discussions

This section is divided into two main parts. In the first part, results from the detection of different breathing patterns are presented and discussed. In the second part, the results of the classification of breathing patterns using various ML algorithms are presented and discussed.

### 4.1. Breathing Pattern Detection

**Figure 5:**
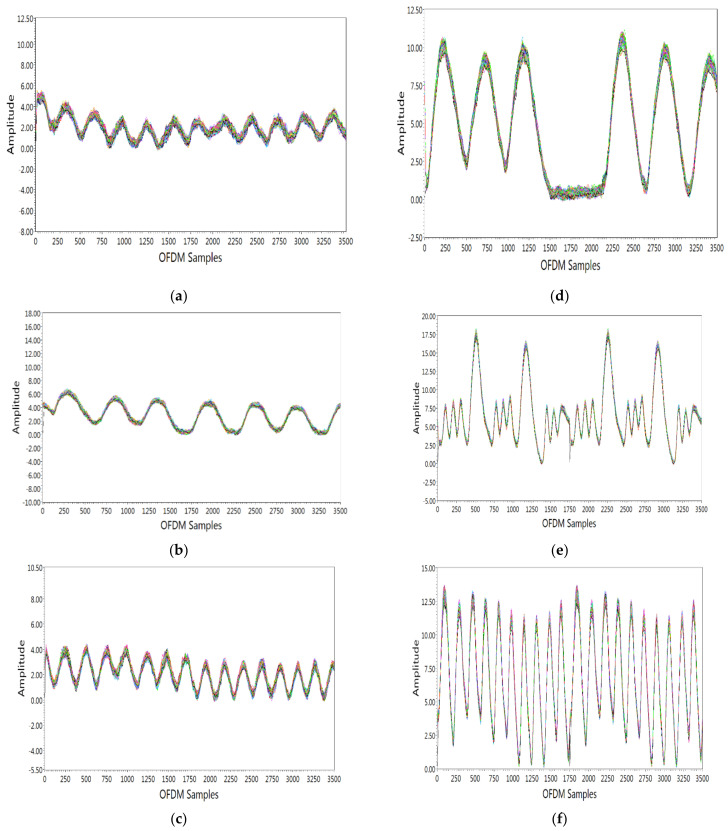
Detection of different abnormal breathing patterns: (**a**) eupnea; (**b**) bradypnea; (**c**)tachypnea; (**d**) biot; (**e**) sighing; (**f**) kussmaul.

In this section, the detection of each breathing pattern using SDR-based RF sensing is described in detail. Each breathing activity was performed five times by five different subjects. Before performing these different breathing activities, each subject was familiarized with the characteristics of all breathing patterns via proper guidance and training. Each subject was asked to practice these breathing patterns for some time. The results were then obtained for six different breathing patterns. The amplitude response of CSI was used to analyze these breathing patterns. Figure 5 shows the amplitude response of all subcarriers for six different breathing patterns. The change in amplitude response was obtained from each activity over 3500 OFDM samples. The results from subject 2 for different breathing activities are shown in Figure 5 for illustration purpose and are discussed below.

- Eupnea is breathing with a normal pattern and rate. Eupnea is usually 12–20 breaths per minute for adults. For this breathing pattern, the subject was requested to breathe normally at a normal rate. From Figure 5a, it is seen that there were 10 breaths per 30 s, which agrees with the breathing patterns shown in Figure 1a.
- Bradypnea is a slow and shallow breath. For this breathing pattern, the subject was requested to breathe more slowly than a normal rate. From Figure 5b, it is seen that there were 6 breaths per 30 s, which agrees with the breathing patterns shown in Figure 1b.
- Tachypnea is a fast and shallow breath. For this breathing pattern, the subject was requested to breathe faster than the normal rate. From Figure 5c, it is seen that there were 13 breaths per 30 s, which agrees with the breathing patterns shown in Figure 1c.
- Biot is a deep breath with gradual periods of no breaths. The subject was requested to perform this breathing pattern. From Figure 5d, it is seen that there were deep breaths followed by no breaths, which agrees with the breathing patterns shown in Figure 1d.
- Sighing is breathing punctuated by frequent deep breathes. The subject was requested to perform this breathing pattern. From Figure 5e, it is seen there was normal breathing punctuated by frequent deep breaths, which agrees with the breathing patterns shown in Figure 1e.
- Kussmaul is a fast and deep breath. The subject was requested to perform this breathing pattern. From Figure 5f, it is seen that there were deep and fast breaths, which agrees with the breathing patterns shown in Figure 1f.

### 4.2. Abnormal Breathing Patterns Classification

This section discusses the different ML algorithms used to classify different breathing patterns. The five-fold cross-validation technique was used on different breathing patterns data. A confusion matrix was used to check the performance of each ML algorithm, as shown in Table 4, because there were six different breathing patterns, there was a total of six predicted and true classes. In the confusion matrix, the columns represent the predicted class, whereas the rows represent the true class of the algorithm. The diagonal cells of the matrix show the cases where the actual class and predicted class are matched. The cell values other than diagonal cells show where the ML algorithm performed poorly. The performance of each ML algorithm was evaluated on the basis of accuracy, prediction speed, and training time, as shown in Table 5. Accuracy was calculated in percentage, prediction speed was measured in observation per second, and training time was calculated in seconds. There were 3650 samples for each breathing activity of 30 s duration, and a total of six different activities; thus, there were a total of 21,900 samples of all breathing activities. Four different ML algorithms were used as classifiers. Among all ML algorithms, the decision tree algorithm shows the best classification accuracy. The parameters and performance detail of each ML algorithm is shown below:

The decision tree algorithm was used with a preset complex tree. The maximum number of splits was set to 100 and the gini diversity index was used as the split criterion. The confusion matrix depicts that a total of 3650 samples were present for each breathing activity, and out of 3650 samples, maximum number of samples were classified correctly. Very few samples were classified incorrectly. This very small classification error occurred between the first two classes, that is, eupnea and bradypnea. The possible reason for this error is that the fundamental difference between eupnea and bradypnea is the breathing rate; eupnea is normal breathing, which is characterized by 12–20 breaths/min, whereas bradypnea is a type of breathing with a slower rate than that of eupnea. The breathing rate in eupnea can be as slow as 12 breaths/min and as fast as 20 breaths/min. Therefore, the breathing of an individual who usually breathes slowly may be similar to another individual’s slow breath. This results in classification errors between eupnea and bradypnea. The overall percentage accuracy using a complex tree was obtained as 99.4%. The ensemble algorithm was used with preset subspace K-Nearest Neighbor (KNN). For this algorithm, the subspace method was used as the ensemble method, and the nearest neighbor was set as the learner type. The confusion matrix shows almost the same results as those of the complex tree algorithm. Maximum samples were classified correctly. However, in this case, classification error occurred between the first three classes, that is, eupnea, bradypnea, and tachypnea. The reason for this is the same as that stated above: a person’s normal breath may be as high as 20 breaths/min, which may be as same as another person’s fast breaths. This results in a classification error between tachypnea and eupnea. The overall percentage accuracy using KNN was obtained as 98.6%. A Support Vector Machine (SVM) algorithm was used with a preset quadratic SVM. A quadratic kernel function is used with an automatic kernel scale. The confusion matrix shows almost the same results as the first two algorithms. Maximum samples were classified correctly. The overall percentage accuracy using the quadratic SVM was obtained as 97.3%. A K-Nearest Neighbor Classifiers (KNN) algorithm was used with a preset coarse KNN. The number of neighbors was set to 100 with Euclidean as the distance metric. The confusion matrix shows almost the same results as those of all other algorithms. Maximum samples were classified correctly. The overall percentage accuracy using coarse KNN was obtained as 94.2%.

## 5. Conclusions

At present, a global pandemic is underway. In this regard, a non-contact SDR platform was developed in this study for the detection of breathing patterns and classification under COVID-19 and non-COVID-19 scenarios. The variations in CSI due to human breathing were utilized to detect different breathing patterns using fine-grained OFDM symbols. Then, ML algorithms were used to accurately classify these patterns. Thus, it can be concluded that SDR-based RF sensing is a suitable solution for the detection and classification of different breathing patterns that relate to certain diseases in an indoor environment. However, this research has a number of limitations. First, this platform can currently be used for a single subject at one time in a controlled and static environment. The second limitation is that the experiments were not performed on real patients. Finally, the third limitation is that this work was not compared with any standard reference. Therefore, the future recommendation of this research work would be to include breathing detection of multiple subjects in a non-static environment, using more advanced algorithms and exploiting the flexibility of the SDR platform. In addition, real-time data collection of patients affected by COVID-19 will be conducted to develop a realistic model, and a comparison of results with a standard reference will also be performed. In future work, the detection capabilities of the platform will also be enhanced by including other breathing patterns, such as cheynestokes and ataxic.

## Figures and Tables

**Table 1:** Causes and description of different abnormal breathing patterns.

Sr. #	Breathing Pattern Type	Description	Causes
1.	Eupnea	Normal breathing pattern and rate	Balanced diet and healthy life
2.	Bradypnea	Slow and shallow breathe	Sleep drugs, metabolic disorder, head injury, stroke
3.	Tachypnea	Fast and shallow breathe	Fever, anxiety, exercise, shock
4.	Biot	Deep breathe with gradual periods of no breaths	Spinal meningitis, head injury
5.	Sighing	Breathing punctuated by frequent deep breathes	Anxiety, dyspnea, and dizziness
6.	Kussmaul	Fast and deep breaths	Renal failure, metabolic acidosis, diabetic ketoacidosis

**Table 2:** Subject details.

Sr. No.	Subject	Age (Years)	Height (cm)	Weight (Kg)	Body Structure
1	Male	28	179	65	Ectomorph
2	Male	31	176	52	Endomorph
3	Male	26	173	76	Endomorph
4	Male	31	177	52	Ectomorph
5	Male	31	174	65	Endomorph

**Table 3:** Statistical feature expressions for correct classification.

Sr. #	Statistical Features	Description	Expression
1	Minimum	Minimum value of data	$Y_{min}=min\left(y_{i}\right)$
2	Maximum	Maximum value of data	$Y_{max}=max\left(y_{i}\right)$
3	Mean	Mean of data	$Y_{m}=\frac{1}{N}\overset{N}{\underset{i=1}{\sum }}y_{i}$
4	Variance	degree of data spread	$Y_{S\;D}=\overset{n}{\underset{i=1}{\sum }}{\left(y_{i}-Y_{m}\right)}^{2}$
5	Standard deviation	Square root of variance	$Y_{v}=\sqrt[{2}]{\frac{1}{N-1}\overset{N}{\underset{i=1}{\sum }}{\left(y_{i}-Y_{m}\right)}^{2}}$
6	RMS	Root mean square data	$Y_{RMS}=\sqrt[{2}]{\frac{1}{N}\overset{N}{\underset{i=1}{\sum }}y_{i}{}^{2}}$
7	Peak-to-peak value	Data fluctuations about the mean	$Y_{p-p}=Y_{max}-Y_{min}\left(i=1,2,\ldots ,N\right)$
8	Kurtosis	Measure of tailedness in data	$Y_{K}=\frac{\frac{1}{K}\sum_{i=1}^{K}{\left(\left|\begin{array}{c} y_{i} \end{array}\right|-Y_{m}\right)}^{4}}{Y_{RMS}{}^{4}}$
9	Skewness	Measure of symmetry in data	$Y_{S}=\frac{\frac{1}{N}\sum_{i=1}^{N}{\left(\left|\begin{array}{c} y_{i} \end{array}\right|-Y_{m}\right)}^{3}}{Y_{RMS}{}^{3}}$
10	Peak factor	Ratio of maximum data value to RMS	$Y_{P}=\frac{max\left(y_{i}\right)}{Y_{RMS}}\;\left(i=1,2,\ldots ,N\right)$
11	Interquartile range	Mid-spread of data	$Y_{IQ}=Q_{3}-Q_{1}$
12	Waveform factor	Ratio of the RMS value to the mean value	$Y_{W}=\frac{Y_{RMS}}{Y_{M}}$
13	FFT	Frequency information about data	$Y_{FFT}=\overset{N}{\underset{n=-N}{\sum }}y\left(n\right)e^{-j\frac{2\pi }{N}nk}$
14	Frequency Min	Minimum Frequency component	$Y_{fmin}=Min\left(Y_{FFT}\right)$
15	Frequency Max	Maximum Frequency component	$Y_{fmax}=Max\left(Y_{FFT}\right)$
16	Spectral Probability	Probability distribution of spectrum	$Y_{SP}=\frac{FFT{\left(d\right)}^{2}}{\sum_{i=-N}^{N}FFT{\left(i\right)}^{2}}$
17	Signal Energy	Measure of energy component	$Y_{SE}=\overset{N}{\underset{n=-N}{\sum }}{\left|p\left(d\right)\right|}^{2}$
18	Spectrum Entropy	Measure of data irregularity	$Y_{H}=\overset{N}{\underset{i=-N}{\sum }}p\left(d\right)\ln\left(p\left(d\right)\right)$

**Table 4:** Confusion matrix of ML algorithms.

Algorithms	Actual/Predicted	Eupnea	Bradypnea	Tachypnea	Biot	Sighing	Kussmaul
Complex Tree	Eupnea	3608	33	0	9	0	0
	Bradypnea	40	3604	0	6	0	0
	Tachypnea	0	0	3632	0	0	18
	Biot	9	3	0	3638	0	0
	Sighing	0	0	0	0	3649	1
	Kussmaul	0	0	4	0	1	3645
Ensemble Subspace KNN	Eupnea	3509	18	59	3	0	1
	Bradypnea	29	3594	23	0	0	4
	Tachypnea	99	37	3505	9	0	0
	Biot	1	0	1	3638	0	4
	Sighing	0	0	0	0	3650	0
	Kussmaul	2	5	1	1	0	3641
Quadratic SVM	Eupnea	3403	185	0	61	1	
	Bradypnea	160	3485	0	5	0	0
	Tachypnea	18	6	3589	18	0	19
	Biot	117	2	0	3531	0	0
	Sighing	0	0	0	0	3650	0
	Kussmaul	0	2	0	4	2	3642
Coarse KNN	Eupnea	3278	137	109	101	0	25
	Bradypnea	85	3436	104	2	0	23
	Tachypnea	195	134	3197	49	0	75
	Biot	46	1	40	3523	10	30
	Sighing	0	0	0	0	3649	1
	Kussmaul	14	51	0	21	20	3544

**Table 5:** Performance of ML algorithms.

Algorithms	Accuracy (%)	Prediction Speed (obs/s)	Training Time (s)
Complex Tree	99.4	~330,000	7.58
Ensemble Subspace KNN	98.6	~12,000	239.40
Quadratic SVM	97.3	~21,000	193.92
Coarse KNN	94.2	~2400	182.18

## Data Availability

Not applicable.
